# Exacerbation of Liver Tumor Metastasis in *twist1a+*/*xmrk+* Double Transgenic Zebrafish following Lipopolysaccharide or Dextran Sulphate Sodium Exposure

**DOI:** 10.3390/ph14090867

**Published:** 2021-08-28

**Authors:** Jeng-Wei Lu, Yuxi Sun, Liang-In Lin, Dong Liu, Zhiyuan Gong

**Affiliations:** 1 Department of Biological Sciences, National University of Singapore, Singapore 117543, Singapore; e0437708@u.nus.edu; 2 Department of Clinical Laboratory Sciences and Medical Biotechnology, National Taiwan University, Taipei 10048, Taiwan; lilin@ntu.edu.tw; 3 Department of Biology, Southern University of Science and Technology, Shenzhen 518055, China; liu@sustech.edu.cn; 4 Department of Laboratory Medicine, National Taiwan University Hospital, Taipei 10048, Taiwan

**Keywords:** dextran sulfate sodium, lipopolysaccharides, liver tumor metastasis, *twist1a*, *xmrk*, double transgenic zebrafish

## Abstract

The poor prognosis for patients with hepatocellular carcinoma (HCC) is related directly to metastasis. The *Twist1* gene encodes for a transcription factor essential to embryogenesis. It has also been shown to promote epithelial-to-mesenchymal transition (EMT), invasion, and metastasis; however, there is currently no in vivo evidence that *Twist1* plays a role in the metastasis of liver tumors. Zebrafish are increasingly being used as an alternative cancer model. In the current study, an adult-stage zebrafish HCC model was used to examine the synergistic effects of *twist1a* and *xmrk*, a well characterized oncogene, during HCC metastasis. We also examined the effects of two inflammatory agents, lipopolysaccharides (LPS) and dextran sulfate sodium (DSS), on the hepatocyte-specific expression of transgenic *twist1a* and *xmrk*. The conditional overexpression of *twist1a* and *xmrk* was shown to promote liver tumor metastasis in zebrafish, resulting in increased apoptosis and cell proliferation as well as tumor maintenance and propagation independent of the inherent EMT-inducing activity of *xmrk*. Exposing *twist1a+*/*xmrk+* transgenic zebrafish to LPS or DSS was shown to promote metastasis, indicating that the overexpression of *twist1a* and *xmrk* led to crosstalk between the signaling pathways involved in EMT. This study provides important evidence pertaining to the largely overlooked effects of signaling crosstalk between *twist1a* and *xmrk* in regulating HCC metastasis. Our results also suggest that the co-expression of *twist1a*/*xmrk* in conjunction with exposure to LPS or DSS enhances HCC metastasis, and provides a valuable in vivo platform by which to investigate tumor initiation and metastasis in the study of liver cancer.

## 1. Introduction

Hepatocellular carcinoma (HCC) is a common cause of death worldwide, particularly in Sub-Saharan Africa and Southeast Asia [1]. HCC is strongly associated with chronic hepatitis B virus (HBV) and hepatitis C virus (HCV) infection, alcohol abuse, long-term exposure to aflatoxin B1, and metabolic diseases [2]. In recent years, researchers have observed increases in HCC incidence and HCC-related mortality [3]. Surgical resection and liver transplantation can achieve favorable treatment outcomes for patients with non-metastatic HCC. These patients are also candidates for palliative local treatments, such as chemoembolization, radiofrequency ablation, and stereotactic radiotherapy [4]. However, for patients with advanced or metastatic HCC, palliative systemic therapy is the only option, and the survival benefits are limited [5]. As a result, HCC remains among the most deadly cancers, with a 5-year survival rate of only 5% [6].

In the event of tumor metastasis, the prognosis is generally poor. This process (referred to as the invasion metastatic cascade) involves a number of events, which must occur is the correct sequence. This process involves the dissemination of cancer cells to anatomically distant organs and subsequent invasion into other tissue microenvironments. Metastasis can then lead to complications and the development of diseases independent of the original tumor [7].

The epithelial-to-mesenchymal transition (EMT) refers to epithelial cells giving up adhesion properties in favor of mesenchymal characteristics [8,9,10]. EMT activation by cancer cells equips them with the ability to leave the primary tumor, migrate throughout the body, and invade distant organs to form metastases [9,11]. Studies have demonstrated that in the process of EMT, apoptosis and oncogene-induced senescence are suppressed, and cancer cells acquire stem cell-like properties [12]. In numerous cancer cell lines, EMT and cancer stem cell properties have been induced through the overexpression of transcription factors, such as the *Twist1*, *Snail*, *Zeb*, and *FoxC* families [9,11,13,14]. *Twist1* is a basic helix-loop-helix (bHLH) transcription factor central to the embryonic development of gastrulation, neurogenesis, and other programs during morphogenesis [9,15]. It is also a prototype EMT regulator [16]. In most adult human tissues, *Twist1* expression is usually undetectable; however, the overexpression of *Twist1* has been reported in tumors of varying origins, such as breast cancer, bladder cancer, glioma, prostate cancer, pancreatic cancer, sarcoma, squamous cell carcinoma, osteosarcoma, and melanoma [15,16,17,18,19]. High *Twist1* expression in cancers has been linked to metastasis and tumor cell invasion. Note that the *Twist1* expression in tumor cells takes place under hypoxic conditions, which stimulate tumor cell dissemination to less hostile environments, presumably through the promotion of EMT [20,21]. *Twist1* overexpression has also been linked to a poor prognosis for survival in patients with colorectal cancer, oral cancer, esophageal squamous cell carcinoma, and renal cell carcinoma [22,23,24,25].

*Twist1* is believed to regulate EMT by downregulating key proteins that maintain epithelial cell characteristics and by upregulating proteins crucial to the mesenchymal phenotype [26]. For example, the overexpression of *Twist1* has been shown to suppress the expression of E-cadherin, an important molecule in cell–cell adhesion, suggesting that *Twist1* promotes tumor metastasis through the induction of EMT pathways [26,27,28]. *Twist1* overexpression is linked to tumor malignancy early in oncogenesis and has been shown to stimulate tumor progression [29]. *Twist1* overexpression has also been linked to self-renewal in mammary epithelial and cancer cell lines, indicating that *Twist1* contributes to tumor stemness [13,14]. In light of findings highlighting the role of *Twist1* in tumorigenesis and metastasis, researchers have recently engineered a tamoxifen-controllable *twist1a* transgenic zebrafish as a novel animal model for the discovery of anti-metastasis drugs targeting the metastatic dissemination of cancer cells [30].

Researchers have yet to determine the extent to which EMT, proliferation, apoptosis, and inflammation in cancer are regulated via *Twist1*, and whether these processes are independent. In the current study, we sought to elucidate these important issues using a zebrafish model of liver tumorigenesis, induced through the overexpression of *xmrk* (activated epidermal growth factor receptor (EGFR) homolog) [31], a well characterized oncogene shown to work synergistically with *Twist1* during tumorigenesis [27]. Liver tumorigenesis can be clearly divided into independent stages, including tumor initiation, progression [32], and metastasis [33]. To date, there have been no reports on the use of an autochthonous model to study the role of *Twist1* overexpression in the initiation and maintenance of liver tumorigenesis.

In this study, we generated an inducible *twist1a+*/*xmrk+* double transgenic zebrafish model. We then induced the expression of *twist1a* against a background of *xmrk* overexpression in various stages of liver tumorigenesis, with the aim of elucidating the functions and roles of *twist1a* in liver tumor initiation, maintenance, proliferation, and malignant progression in adult-stage zebrafish. We also exposed *twist1a+*/*xmrk+* double transgenic zebrafish to two inflammatory agents, lipopolysaccharides (LPS) or dextran sulfate sodium (DSS), and examined the effects on liver tumor metastasis.

## 2. Results

### 2.1. Twist1a+ Transgenic Zebrafish, Diagram of Experimental Design, and Schedules of Specimen Collection from Long-Term Treatment

Following treatment with Dox, *Twist1a* transgene expression was demonstrated in F3 zebrafish by visualizing mCherry in the hepatocytes of seven dpf larva and five mpf adult zebrafish (Supplementary Figure S1A). To compare tumor growth and metastasis development between *twist1a+* and *twist1a+/xmrk+* double transgenic zebrafish, *twist1a+/xmrk+* zebrafish were treated with Dox and 4-OHT or treated with Dox and 4-OHT exposed to LPS or DSS for up to 8 weeks. Samples were collected weekly for histological examination (Supplementary Figure S1B).

### 2.2. Phenotype of Liver Tumor Metastasis Induced through the Weak Induction of twist1a and xmrk in Transgenic Zebrafish

**Figure 1:**
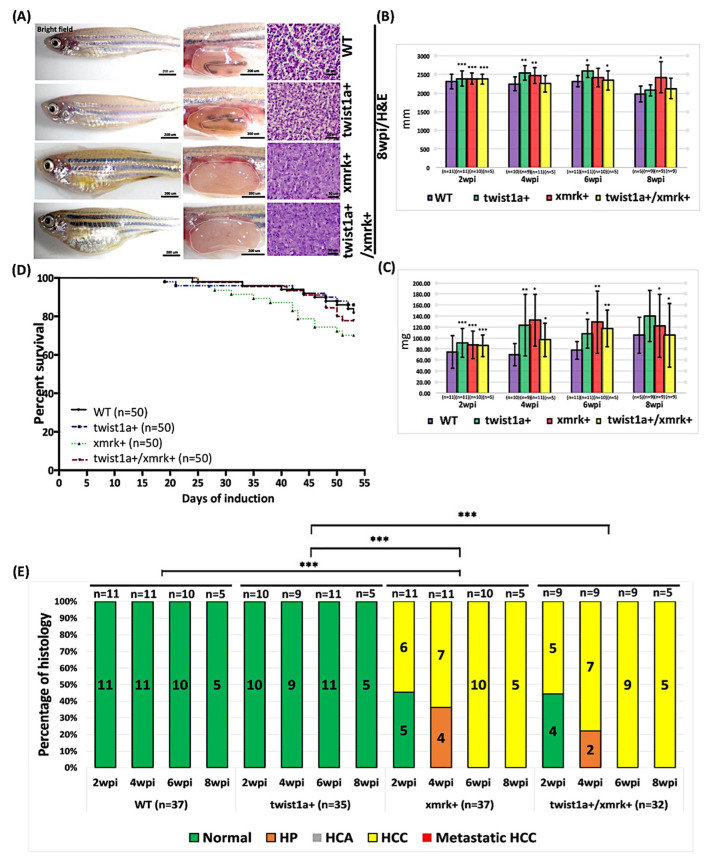
Induction of tumor metastasis in *twist1a+*/*xmrk+* transgenic zebrafish via treatment with Dox in low concentrations. *Twist1a*+, *xmrk*+, and *twist1a+*/*xmrk+* transgenic zebrafish as well as wild-type controls were treated with 30 μg/mL Dox and 0.5 μg/mL 4-OHT starting from 3 mpf. Samples were collected at 2, 4, 6, and 8 wpi. (**A**) Representative images of transgenic zebrafish at 8 wpi. The left column displays the external appearance, the middle column shows internal abdominal organs with the livers outlined, and the right column depicts H&E staining of liver sections. Scale bar: 50 or 200 μm. Compared with the wild-type group, the body size, (**B**) body length, and (**C**) weight of most transgenic zebrafish differed significantly at 2, 4, 6, and 8 wpi. (**D**) Kaplan–Meier survival curves of days post-induction plotted against percent survival until 8 wpi. (**E**) Histological examination confirmed that *xmrk*+ and *twist1a+*/*xmrk+* transgenic zebrafish developed HCC at 2, 4, 6, and 8 wpi, whereas normal liver histology was observed in all *twist1a*+ and wild-type siblings. Differences among variables were assessed using Student’s *t*-tests or one-way ANOVA. Statistical significance: * *p* < 0.05, ** *p* < 0.01, *** *p* < 0.001.

We then examined long-term liver tumor metastasis in *xmrk+*, *twist1a+*, and *twist1a+*/*xmrk+* transgenic zebrafish and their non-transgenic wild-type siblings by treating the animals with 30 μg/mL Dox and 0.5 μg/mL 4-OHT to induce *xmrk* and *twist1a*, respectively. Samples were collected at 2, 4, 6, and 8 wpi for examination of tumor status and metastasis. Under low-concentration Dox and 4-OHT treatment, the *xmrk+* and *twist1a+*/*xmrk+* transgenic zebrafish displayed enlarged abdomens and obvious liver overgrowth, compared with the control groups (wild-type and *twist1a+* zebrafish) (Figure 1A), as early as 2 weeks after induction. A hematoxylin and eosin (H&E) examination of the samples from 2, 4, 6, and 8 wpi revealed the progression of liver tumors from predominantly hyperplasia to HCC in all the zebrafish. The transgenic zebrafish (i.e., *xmrk+*, *twist1a+*, and *twist1a+*/*xmrk+*) presented a body size that was significantly larger than that of wild-type zebrafish (Figure 1B,C). Despite abnormal findings in the liver, we did not observe a significant difference between the *xmrk+* or *twist1a+*/*xmrk+* transgenic zebrafish and the wild-type controls in terms of mortality (Figure 1D). At 4 wpi, some of the *xmrk+* and *twist1a+*/*xmrk+* transgenic zebrafish presented evidence of hyperplasia (HP) (4/11, 36.36%; 2/9, 22.22%, respectively) and hepatocellular carcinoma (HCC) (7/11, 63.64%; 7/9, 77.78%, respectively). At 6 and 8 wpi, all the *xmrk+* and *twist1a+*/*xmrk+* transgenic zebrafish developed severe HCC (6 wpi: 10/10, 100%; 9/9, 100%, respectively; 8 wpi: 5/5, 100%; 5/5, 100%, respectively). No ectopic or metastatic tumors were observed during the 8 weeks of tumor growth after treatment with low concentrations of Dox and 4-OHT (Figure 1E).

### 2.3. High-Concentration Dox Treatment Induced Liver Tumor Metastasis in twist1a+/xmrk+ Double Transgenic Zebrafish

**Figure 2:**
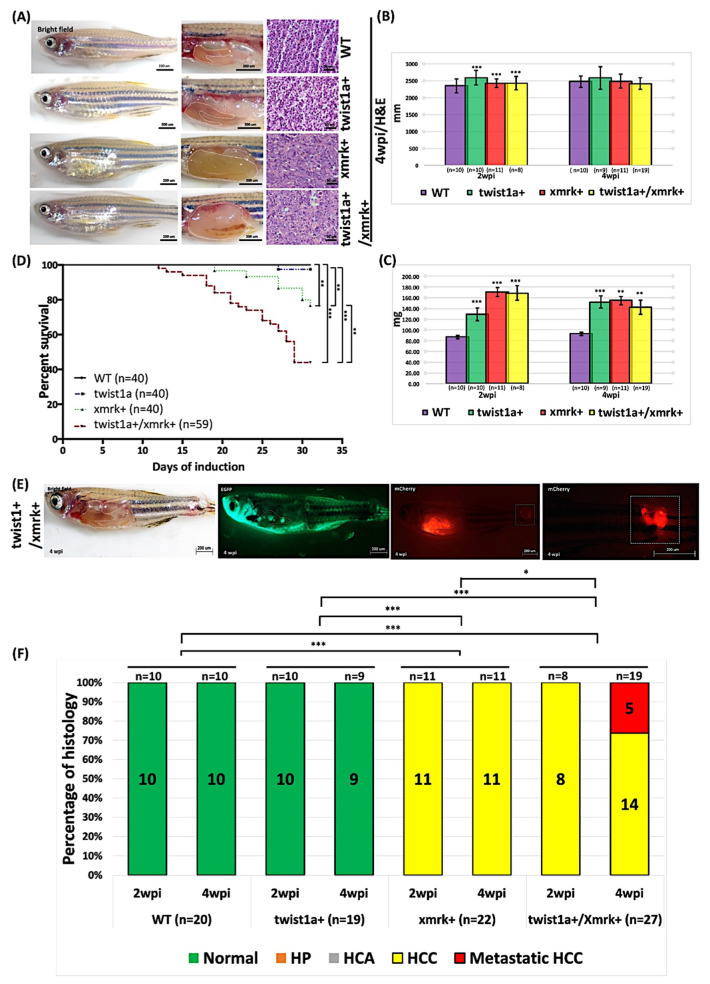
Induction of tumor metastasis in *twist1a+*/*xmrk+* transgenic zebrafish via treatment with Dox in high concentrations. *Twist1a*+, *xmrk*+, and *twist1a+*/*xmrk+* transgenic zebrafish as well as wild-type control were treated with 60 μg/mL Dox and 1 μg/mL 4-OHT starting from 3 mpf. Samples were collected at 2 and 4 wpi. (**A**) Representative images of transgenic zebrafish at 4 wpi. The left column displays external appearance, the middle column shows internal abdominal organs with the livers outlined, and the right column depicts H&E staining of liver sections. Scale bar: 50 or 200 μm. Compared with the wild-type group, (**B**) the body lengths of transgenic zebrafish differed significantly at 2 wpi but not at 4 wpi, whereas (**C**) the body weights of transgenic zebrafish differed significantly at 2 and 4 wpi. (**D**) Kaplan–Meier survival curves of days post-induction plotted against percent survival to 4 wpi. (**E**) Immunofluorescence analysis of liver tumor metastasis in *twist1a*+/*xmrk*+ transgenic zebrafish at 4 wpi. (**F**) Histological examination confirmed that *xmrk*+ and *twist1a+*/*xmrk+* transgenic zebrafish developed HCC or metastatic HCC at 2 and 4 wpi, whereas normal liver histology was observed in all *twist1a*+ and wild-type siblings. Differences among variables were assessed using Student’s *t*-tests or one-way ANOVA. Statistical significance: * *p* < 0.05, ** *p* < 0.01, *** *p* < 0.001.

All of the transgenic zebrafish (*xmrk+*, *twist1a+*, and *twist1a+*/*xmrk+*) and their non-transgenic wild-type siblings were treated with 60 μg/mL Dox and 1 μg/mL 4-OHT for 4 weeks to examine the effects of *twist1a* and *xmrk* (at elevated concentrations) on liver tumorigenesis and metastasis. The *xmrk+* and *twist1a+*/*xmrk+* transgenic zebrafish developed enlarged abdomens and obvious liver overgrowth at 2 and 4 wpi (Figure 2A). Furthermore, the body size was significantly larger than that of the wild-type control zebrafish (Figure 2B,C) and mortality was significantly lower (Figure 2D). The *xmrk+* and *twist1a+*/*xmrk+* animals developed HCC at 2 and 4 wpi (2 wpi: 11/11, 100%; 8/8, 100%, respectively; 4 wpi: 11/11, 100%; 14/19, 73.68%, respectively), and the *twist1a+*/*xmrk+* transgenic zebrafish presented evidence of liver metastasis at 4 wpi (5/19, 26.32%) (Figure 2E,F).

Given the elevated mortality in the *xmrk+* and *twist1a+*/*xmrk+* transgenic zebrafish under higher *xmrk* and *twist1a* induction, we examined the effects of *xmrk* over a range of induction values. In this experiment, the *xmrk+* and *twist1a+*/*xmrk+* transgenic zebrafish were treated with 60 μg/mL Dox for three weeks, at which point the dose was reduced to 30 μg/mL Dox for a further three weeks. Note that 1 μg/mL 4-OHT was maintained throughout the course of the experiment to sustain the induction of *twist1a*. We adopted a treatment period of six weeks to balance tumor growth against zebrafish survival. Liver enlargement was more pronounced in the *twist1a+*/*xmrk+* transgenic zebrafish than in the *xmrk+* transgenic zebrafish (Supplementary Figure S2A). The body size was also significantly larger (Supplementary Figure S2B,C) and mortality was significantly higher (Supplementary Figure S2D). Both the *xmrk* and *twist1a+*/*xmrk+* transgenic zebrafish presented evidence of HCC at 5 and 6 wpi (5 wpi: 10/10, 100%; 10/10, 100%, respectively; 6 wpi: 9/10, 90%; 8/10, 80%, respectively). Evidence of liver tumor metastasis was observed in the *twist1a+*/*xmrk+* transgenic zebrafish at 5 and 6 wpi (metastatic HCC: 1/10, 10%; 2/10, 20%, respectively) (Supplementary Figure S1E).

### 2.4. Expression of Liver Markers fabp10a and tfa in Primary and Metastatic Liver Tumors Tissues in twist1a+/xmrk+ Double Transgenic Zebrafish

**Figure 3:**
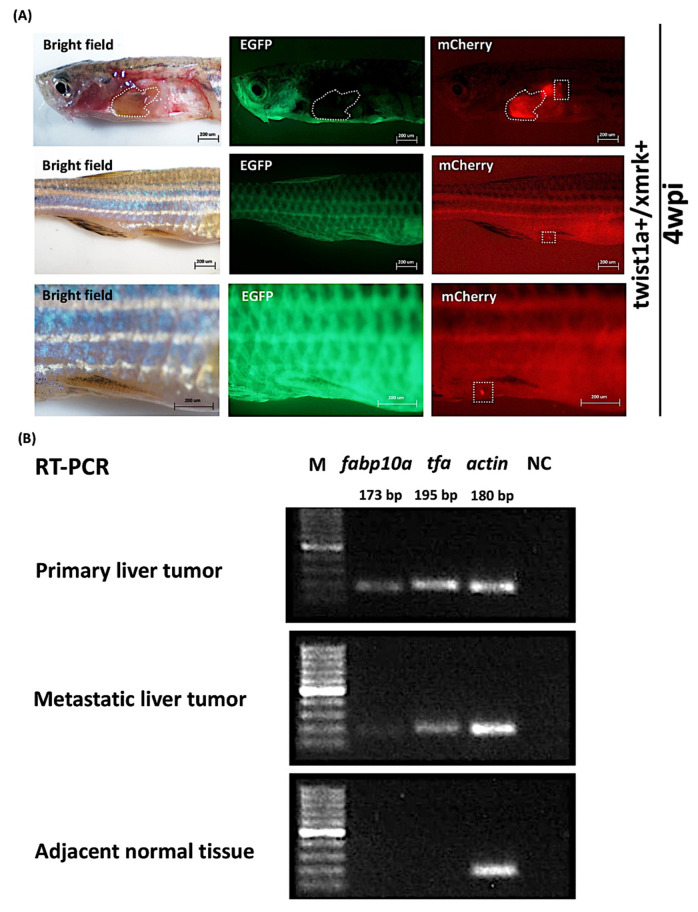
Expression of liver markers *fabp10a* and *tfa* in primary and metastatic liver tumor tissues from *twist1a+*/*xmrk+* double transgenic zebrafish. *Twist1a+*/*xmrk+* transgenic zebrafish were treated with 60 μg/mL Dox and 1 μg/mL 4-OHT. (**A**) mCherry immunofluorescence analysis of *twist1a*+/*xmrk*+ liver tumor metastasis at 4 wpi. Scale bar: 200 μm. (**B**) Results of semiquantitative RT-PCR showing the expression of *fabp10a* and *tfa* in primary tumor, metastatic liver tumor, and adjacent normal tissues. *Actin* and non-template, respectively, served as an internal control and negative control.

After four weeks of treatment with 60 μg/mL Dox and 1 μg/mL 4-OHT, the immunofluorescence of *twist1a+*/*xmrk+* transgenic zebrafish revealed evidence of tumor metastasis (Figure 3A). Tissue samples were collected from the primary liver tumor and metastatic tumor as well as adjacent normal tissues to determine the origin of the metastases. We examined the expression of *fabp10a* and *tfa* RNA (two zebrafish liver markers) in the various tissues using a semiquantitative RT-PCR. Both zebrafish liver markers were expressed primarily in the primary and metastatic liver tumor tissue, thereby confirming that the origin of the metastatic tumors was indeed the liver (Figure 3B). Neither *fabp10a* nor *tfa* RNA was observed in the adjacent normal tissues. *Actin* and a non-template sample, respectively, served as an internal control negative control.

### 2.5. Co-Expression of twist1a and xmrk Significantly Increased Apoptosis and Cell Proliferation in the Hepatocyte Cells of Double Transgenic Zebrafish

**Figure 4:**
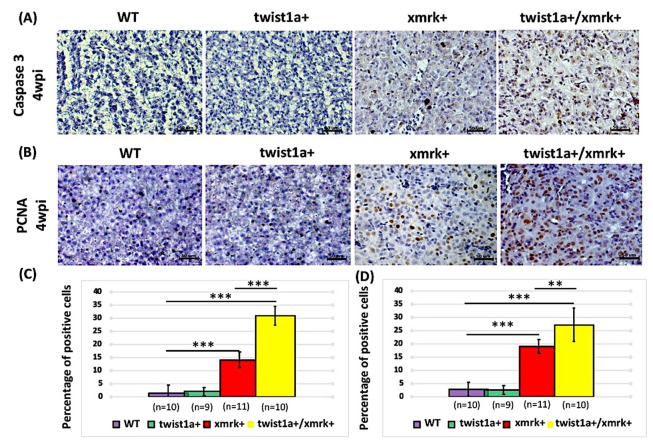
Assessment of apoptosis and cell proliferation in instances of HCC progression in *twist1a+/xmrk+* double transgenic zebrafish. Immunohistochemical staining was performed on liver paraffin sections from wild-type, *twist1a*+, *xmrk*+, and *twist1a+/xmrk+* zebrafish. (**A**) Caspase-3 staining for apoptosis; and (**B**) PCNA staining for proliferation at 4 wpi. Scale bar: 50 μm. Quantification of the percentage of cells testing positive for (**C**) caspase-3 and (**D**) PCNA. Differences among the variables were assessed using Student’s *t*-tests. Statistical significance: ** *p* < 0.01, *** *p* < 0.001.

The main hallmarks of tumorigenesis include cell cycle control and abnormal cell apoptosis [34]; therefore, we sought to determine whether the liver tumorigenesis and metastasis observed in the *twist1a+*/*xmrk+* transgenic zebrafish were a consequence of aberrant cell cycle control and cell apoptosis. Caspase-3 staining for apoptotic cells and PCNA staining for proliferative cells were performed in zebrafish treated with 60 μg/mL Dox and 1 μg/mL 4-OHT (Figure 4A,B). Overall, only a small number of apoptotic cells were observed in non-oncogenic livers in wild-type zebrafish. The number of apoptotic cells in the *xmrk+* and *twist1a+*/*xmrk+* transgenic zebrafish was significantly higher. In fact, the co-induction of *twist1a* and *xmrk* resulted in a 31% increase in the number of apoptotic cells (Figure 4A,C). These findings are consistent with those of our previous research on HCC development in other oncogene transgenic zebrafish [31,35]. Remarkably, the percentage of apoptotic cells was higher in transgenic zebrafish with *twist1a+*/*xmrk+* than in the *xmrk+* induction group (Figure 4C). Other studies have also reported that *EGFR* and *Kras* oncogenes can induce apoptosis through Ras signaling [36,37].

The number of proliferating cells was significantly higher in the *xmrk+* and *twist1a+*/*xmrk+* transgenic zebrafish than in the wild-type control zebrafish. The number of proliferating cells was 27% higher in the *twist1a+*/*xmrk+* transgenic zebrafish (Figure 4B,D); however, the difference was less pronounced in the *xmrk+* transgenic zebrafish (Figure 4D). Note that the extent of apoptosis in individual *twist1a+*/*xmrk+* transgenic zebrafish did not necessarily exceed that of individual *xmrk+* transgenic zebrafish, which suggests that metastatic changes in liver tumors can be attributed primarily to cell proliferation.

### 2.6. Twist1a Expression Activates EMT Pathway via E-cadherin and Vimentin

**Figure 5:**
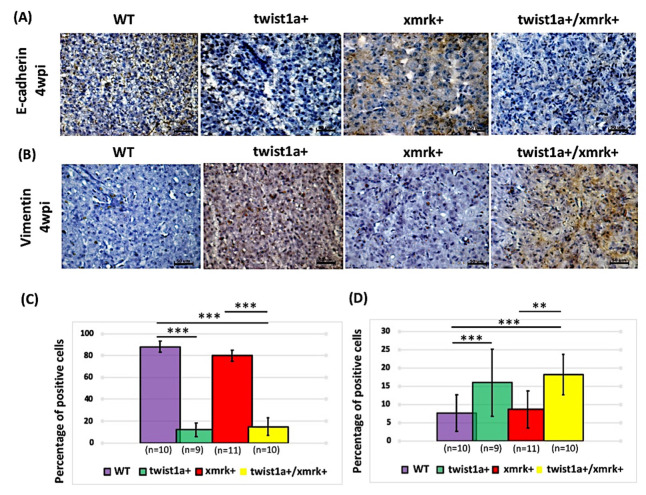
Assessment of E-cadherin and vimentin staining in instances of HCC progression in *twist1a+/xmrk+* double transgenic zebrafish. Immunohistochemical staining was performed on liver paraffin sections from WT, *twist1a+*, *xmrk+*, and *twist1a+/xmrk+* zebrafish at 4 wpi. Staining of markers of EMT activation: (**A**) E-cadherin and (**B**) vimentin. Scale bar: 50 μm. Quantification of the percentage of cells testing positive for (**C**) E-cadherin and (**D**) vimentin. Differences among the variables were assessed using Student’s *t*-tests. Statistical significance: ** *p* < 0.01, *** *p* < 0.001.

The expression of E-cadherin and vimentin are the main hallmarks of EMT during cancer metastasis [9,10]. We performed immunohistochemical staining for E-cadherin and vimentin to determine whether the liver tumor metastasis observed in the *twist1a+*/*xmrk+* transgenic zebrafish was a consequence of aberrant metastasis in the liver (Figure 5A,B). The *twist1a+*/*xmrk+* transgenic zebrafish presented E-cadherin levels lower than those of the wild-type control or *xmrk+* zebrafish with corresponding higher vimentin levels at 4 wpi. The quantification results revealed an 18% decrease in E-cadherin expression in the *twist1a+*/*xmrk+* transgenic zebrafish (Figure 5A,C) and a 17% increase in vimentin expression (Figure 5B,D). This suggests that the co-expression of *twist1a* and *xmrk* triggers crosstalk along the EMT pathway and contributes to the liver tumor metastasis observed in this group of zebrafish.

### 2.7. Exposure to DSS or LPS Induces Gut and Liver Inflammation in lyz:DsRed and mpeg1:mCherry Transgenic Zebrafish Larvae

**Figure 6:**
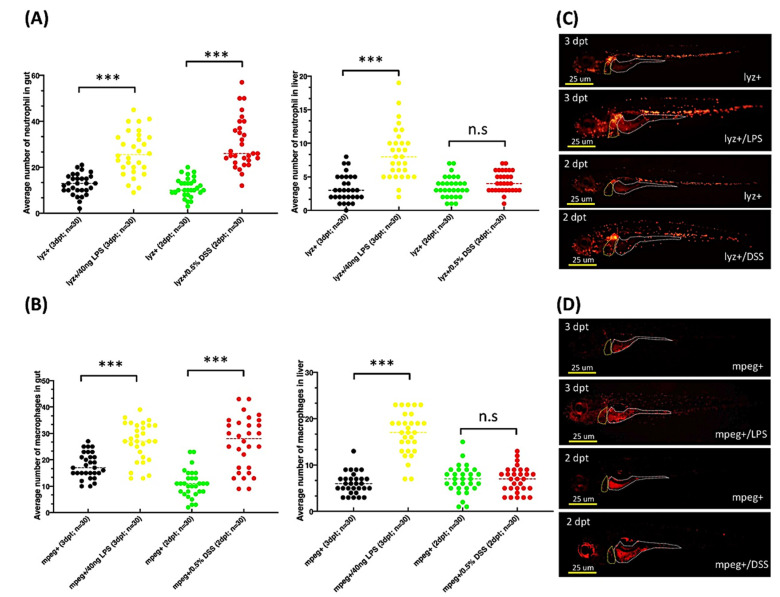
Increases in the numbers of neutrophils and macrophage in the gut and liver of *lyz*+/LPS, *lyz*+/DSS, *mpeg1*+/LPS, and *mpeg1*+/DSS zebrafish larvae exposed to LPS or DSS. Quantification (via fluorescence) of the number of cells in the gut and liver of zebrafish larvae testing positive for (**A**) neutrophils *(lyz*+) or (**B**) macrophages (*mpeg1*+) accompanied by representative fluorescence images of (**C**) neutrophils and (**D**) macrophages. Differences among the variables were assessed using Student’s *t*-tests. Statistical significance: *** *p* < 0.001. Scale bar: 25 μm.

Macrophages and neutrophils are the most abundant immune cells that infiltrate tumors, and both have been implicated in the development of HCC [38,39,40]. We evaluated the effects of elevated macrophage and neutrophil levels on HCC in the *xmrk+* and *twist1a+*/*xmrk+* transgenic zebrafish by determining whether inflammation could be induced by LPS or DSS in zebrafish larvae. This was achieved using Tg(lyz:DsRed, *lyz*+) and Tg(mpeg1:mCherry, *mpeg1*+) zebrafish to, respectively, measure the presence of neutrophils and macrophages. Four-day-old zebrafish larvae were treated with 40 ng/mL LPS or 0.5% DSS (i.e., *lyz*+/LPS, *lyz*+/DSS, *mpeg1*+/LPS, and *mpeg1*+/DSS) for 2 or 3 days. *Lyz*+ and *mpeg1*+ zebrafish larvae without exposure to inflammatory agents served as the controls. All the larvae in each group underwent imaging, whereupon the numbers of neutrophils and macrophages were quantified via immunofluorescence (Figure 6A–D). The exposure to LPS or DSS significantly increased the number of neutrophils and macrophages in the gut in the *lyz*+/LPS, *lyz*+/DSS, *mpeg1*+/LPS, and *mpeg1*+/DSS larvae, compared with the *lyz*+ and *mpeg1*+ control larvae. In the liver, significant increases in the number of neutrophils and macrophages were also observed following LPS exposure in the *lyz*+/LPS and *mpeg1*+/LPS zebrafish larvae; however, we did not observe any changes in the number of these immune cells following exposure to DSS (Figure 6A,B).

### 2.8. Liver Tumor Phenotypes Induced by Sustained Expression of xmrk and Exposure to LPS or DSS in Transgenic Zebrafish Larvae and Adult Transgenic Zebrafish

**Figure 7:**
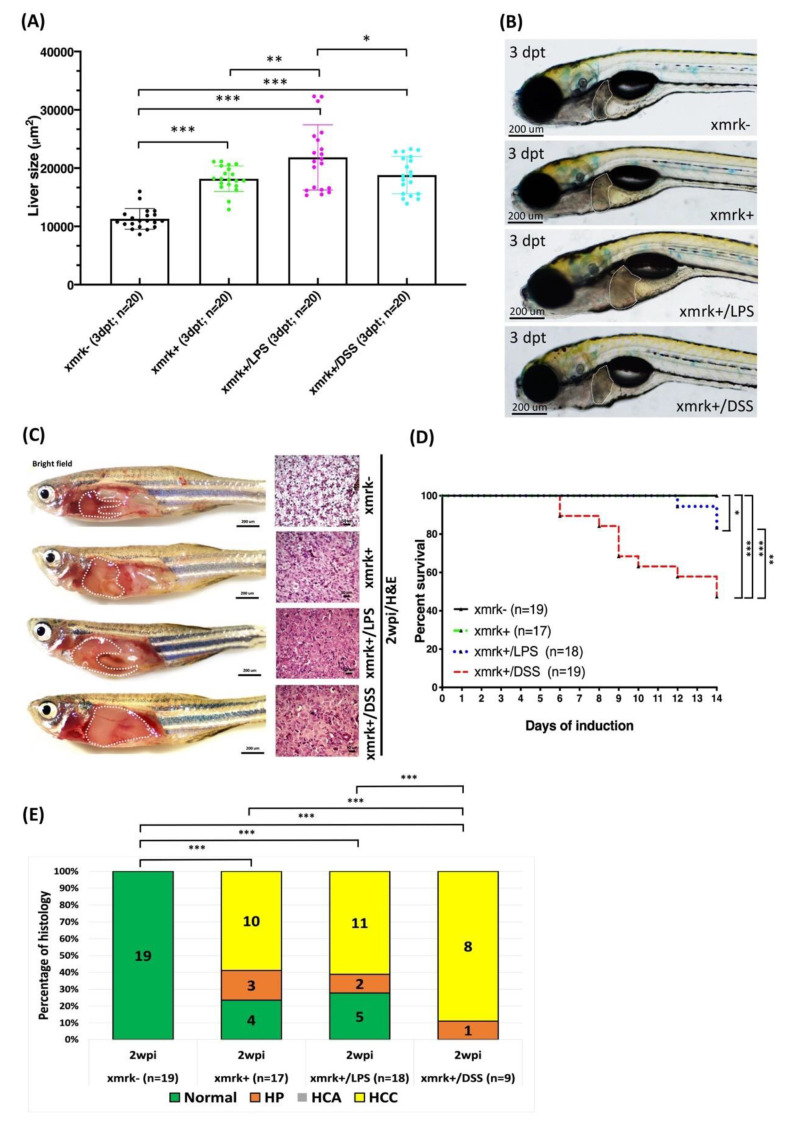
Exposure to LPS or DSS exacerbated the increase in liver size and HCC progression in *xmrk*+ zebrafish. (**A**) Quantification and (**B**) representative images of liver size in *xmrk*+, *xmrk*+/LPS, and *xmrk*+/DSS zebrafish larvae as well as *xmrk*- siblings at 3 dpt. (**C**) *xmrk*+, *xmrk+*/*LPS,* and *xmrk+*/*DSS* transgenic zebrafish as well as *xmrk*- control transgenic zebrafish were treated at 4 mpf with 20 μg/mL Dox plus 40 ng/mL LPS or 0.5% DSS, with samples taken at 2 wpi. The left column shows internal abdominal organs with the livers outlined, and the right column depicts H&E staining of liver sections. Scale bar: 50 or 200 μm. (**D**) Kaplan–Meier survival curves showing days post-induction plotted against percentage survival to 2 wpi. (**E**) Histological examination confirmed that *xmrk*+, *xmrk*+/LPS, and *xmrk+/DSS* transgenic zebrafish developed HCC at 2 wpi, compared to normal liver histology in *xmrk-* siblings. Differences among the variables were assessed using Student’s *t*-tests or one-way ANOVA. Statistical significance: * *p* < 0.05, ** *p* < 0.01, *** *p* < 0.001.

We evaluated the effects of exposure to LPS or DSS on liver tumor progression following the short-term or long-term induction of *xmrk* in the *xmrk+* transgenic zebrafish, which were treated with 20 μg/mL Dox alone or in conjunction with 20 μg/mL Dox and exposed to 40 ng/mL LPS or 0.5% DSS. Note that the *xmrk-* siblings without Dox treatment served as the controls. Following short-term induction, liver size was significantly larger in the *xmrk*+, *xmrk*+/LPS, and *xmrk*+/DSS larvae than in the *xmrk*- control larvae (Figure 7A,B).

Following long-term induction, the *xmrk+* transgenic zebrafish were treated with 20 μg/mL Dox alone or 20 μg/mL Dox and 40 ng/mL LPS or 0.00625% DSS. Note that the *xmrk-* siblings without Dox treatment served as the controls. At 2 wpi, samples were collected for the assessment of tumor status. The *xmrk*+, *xmrk*+/LPS, and *xmrk*+/DSS transgenic zebrafish exhibited enlarged abdomens (compared with the *xmrk*- control group) and obvious signs of liver overgrowth. A H&E examination revealed that following *xmrk* induction, the liver phenotype progressed from predominantly normal to HCC (Figure 7C). A significant increase in mortality was also observed in the *xmrk*+/LPS and *xmrk*+/DSS transgenic zebrafish, compared with the *xmrk*+ transgenic zebrafish and the *xmrk*- controls (Figure 7D). Histological analysis of the *xmrk+*, *xmrk+/LPS*, and *xmrk+/DSS* transgenic zebrafish revealed a combination of the normal liver phenotype (4/17, 23.53%; 5/18, 27.78%; and 0/9, 0%, respectively), hyperplasia (3/17, 17.65%; 2/18, 11.11%; and 1/9, 11.11%, respectively), and hepatocellular carcinoma (10/17, 58.82%; 11/18, 61.11%; and 8/9, 88.89%, respectively), whereas the *xmrk*- controls all presented the normal liver phenotype (19/19, 100%). The HCC status was more severe in the *xmrk+/DSS* than in the *xmrk+* or *xmrk+/LPS* transgenic zebrafish (Figure 7E).

### 2.9. Exposure to LPS or DSS Exacerbated Liver Tumor Metastasis in Hepatocyte-Specific Expression of twist1a+/xmrk+ Double Transgenic Zebrafish

**Figure 8:**
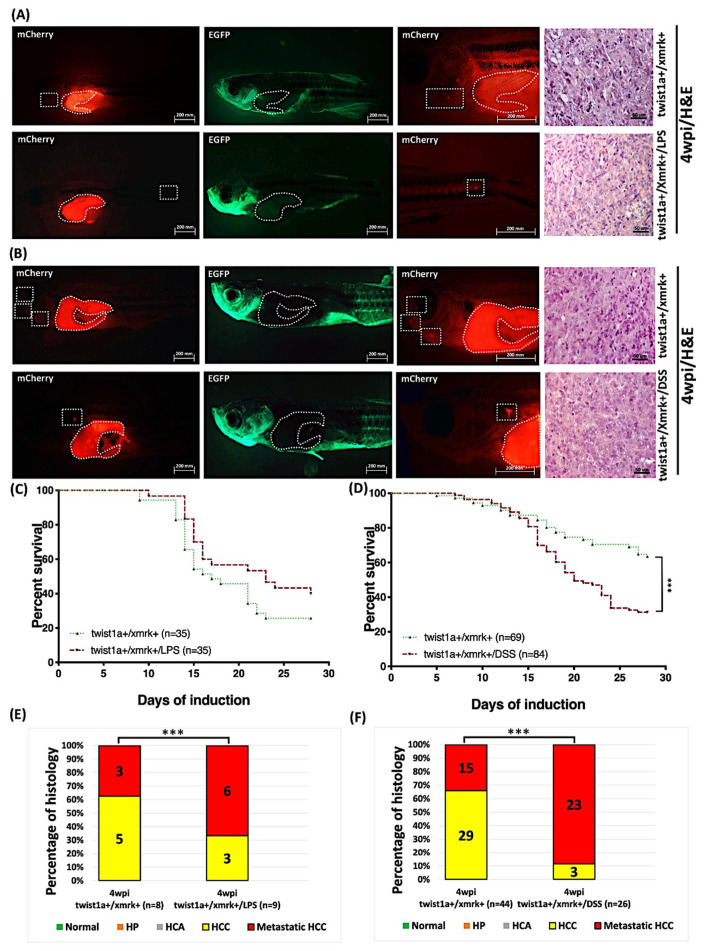
Induction of tumor metastasis in *twist1a+*/*xmrk+* transgenic zebrafish via Dox and 4-OHT treatment under exposure to LPS or DSS. *Twist1a+*/*xmrk+* transgenic zebrafish were treated with 60 μg/mL Dox and 1 μg/mL 4-OHT starting from 4 mpf with sampling performed at 4 wpi. (**A**,**B**) Immunofluorescence analysis of *twist1a*+/*xmrk*+ liver tumor metastasis at 4 wpi with corresponding H&E histological examination of liver sections. Scale bar: 50 or 200 μm. (**C**,**D**) Kaplan–Meier survival curves showing days post-induction plotted against percentage survival to 4 wpi. (**E**,**F**) Histological examination confirmed that at 4 wpi, all *twist1a+*/*xmrk+* transgenic zebrafish developed HCC or metastatic HCC. Differences among the variables were assessed using one-way ANOVA. Statistical significance: *** *p* < 0.001.

Our findings revealed that the simultaneous induction of *xmrk+* and *twist1a+* under exposure to inflammatory agents can enhance liver tumorigenesis and metastasis. Thus, we examined the effects on liver tumor metastasis in *twist1a+*/*xmrk+* transgenic zebrafish exposed to LPS or DSS in the adult stage. At 4 mpf, the *twist1a+*/*xmrk+* zebrafish were treated with 20 μg/mL Dox and 1 μg/mL 4-OHT and exposed to 40 ng/mL LPS or 0.00625% DSS for 4 weeks. The immunofluorescence analysis revealed evidence of tumor metastasis (Figure 8A,B). In terms of mortality, a substantial number of zebrafish in all the groups began to succumb from approximately 10 dpi. No significant difference in mortality was observed between the *twist1a+*/*xmrk+* transgenic zebrafish exposed to LPS and the corresponding unexposed *twist1a+*/*xmrk+* control zebrafish (Figure 8C). Nonetheless, mortality was significantly higher among the *twist1a+*/*xmrk+* zebrafish exposed to DSS than among the unexposed *twist1a+*/*xmrk+* controls (Figure 8D). Furthermore, we observed that at 4 wpi, the incidence of liver tumor metastasis was significantly higher in the *twist1a*+/*xmrk*+/LPS and *twist1a*+/*xmrk*+/DSS zebrafish (6/9, 66.67%; and 23/26, 88.46%, respectively) than in the two unexposed *twist1a*+/*xmrk*+ control groups (3/8, 37.50%; and 15/44, 34.10%, respectively) (Figure 8E,F).

## 3. Discussion

HCC involves a multi-stage alteration in gene expression involving cell proliferation, invasion, and metastasis. Recent advances in surgical techniques have led to significant improvements in local tumor control; however, the prognosis of patients with metastatic HCC remains poor [41]. The dysregulation of human *Twist1* has been reported in HCC and other cancers, and research suggests that *Twist1* plays an important role in promoting the invasion and metastasis of HCC and intrahepatic cholangiocarcinoma [42,43]. Moreover, in a mouse model of metastatic breast tumor, *twist1* has been identified among the most up-regulated genes [26].

Zebrafish is an excellent model by which to investigate the mechanisms underlying metastasis in human cancers [44]. It can also serve as a tool for the screening of therapeutic drugs [2,30,31,45,46]. In the current study, we utilized transgenic zebrafish in the larvae and adult stages to elucidate the processes of liver tumorigenesis and metastasis. We hypothesized that there are additional EMT-related genes (e.g., *twist1a*) that are independent of the primary *xmrk* oncogene, but that can have cumulative effects on tumor progression. In this study, we performed long-term tumor induction for up to 8 weeks (Figure 1 and Figure 2). The hepatocyte-specific overexpression of *twist1a* and *xmrk* in the liver of zebrafish was shown to accelerate apoptosis (Figure 4A,C) and cell proliferation (Figure 4B,D), with concomitant hepatocyte transformations by 4 wpi, including liver overgrowth, HP, HCC, and metastatic HCC (Figure 2 and Figure 3).

The overexpression of *twist1a* was associated with a reduction in the expression of E-cadherin (Figure 5A,C) and an increase in the expression of vimentin (Figure 5B,D), both of which are involved in the promotion of EMT. One clinical study reported that *Twist1* was indicative of tumor cell EMT and endothelium-dependent angiogenesis in HCC [47], suggesting that the activation of the *twist1a* gene could be mediated via regulation of the EMT pathway. By contrast, *Twist1* has been shown to prevent oncogene-induced apoptosis and/or senescence, while decreasing Ras and Myc expression via the repression of the p16 and/or the p19ARF/p53 pathways. The *Twist1* was shown to be essential to the initiation and maintenance of p53-deficient cancer stem cells in a *KRas*^D12^ p53-deficient mouse model. That study identified both p53-dependent and p53-independent roles for twist1 in tumor initiation, proliferation, apoptosis, and propagation [12]. In mice, the impaired expression of E-cadherin was found to promote hepatocellular carcinogenesis [48], and the dysregulation of E-cadherin was also identified in a number of transgenic mouse models of liver cancer [49]. In HCC patients, a decrease in the expression of E-cadherin is indicative of poor prognosis [50]. Moreover, the accelerated autophagic degradation of E-Cadherin via sirtuin 6, a protein deacetylase to promote EMT in HCC [51]. In in vitro and in vivo studies of breast cancer, the repression of E-cadherin expression occurs involving the direct binding of Twist1 to the E-cadherin promoter, such that the downregulation of E-cadherin attenuates cell–cell adhesion and enhances migration and invasion [26].

Vimentin plays important roles in regulating the migration of many cell types [52]. Impaired cell migration has been demonstrated through the recirculation of endocytic cell adhesion receptors to the plasma membrane by vimentin as well as through the disruption of vimentin function [53]. Previous studies have reported the overexpression of vimentin in cases of breast cancer and HCC metastasis [54,55]. The overexpression of vimentin has also been shown to increase integrin traffic, migration, and invasion in a vimentin-negative MCF7 breast cancer cell line [53,56]. In a number of triple-negative breast cancers, vimentin expression has been identified as a marker of basal-like breast cancer cells associated with poor prognosis [57]. Serum vimentin can serve as a surrogate marker for small HCC tumors [58]. Furthermore, vimentin is a potential therapeutic target in a sorafenib-resistant HepG2 cell line [59]. In the current study, we observed that *twist1a* not only maintains but in fact accelerates *xmrk*-induced liver tumorigenesis and metastasis. This finding is consistent with previous studies on other types of cancers, such as those of the skin, bone, and lung [12,60,61].

In the tumor microenvironment, macrophages and neutrophils are the most abundant immune cells that infiltrate tumors [38,39]. As such, macrophages and neutrophils also play a key tumor support role in HCC [40]. Zebrafish models have been used to study the effects of LPS or DSS on inflammation [62,63]. In the current study, we confirmed the following: (1) LPS or DSS can induce inflammation by recruiting neutrophils and macrophages (Figure 6); (2) The effects of LPS or DSS exposure are enhanced when combined with *xmrk* expression (Figure 7); and (3) These effects stimulate the immune system, resulting in accelerated tumor metastasis in *twist1a+*/*xmrk+* double transgenic zebrafish (Figure 8). In the adult-stage *twist1a+*/*xmrk+* zebrafish, the hepatocyte-specific co-expression of *twist1a* and *xmrk* coupled with LPS (Figure 8A,E) or DSS (Figure 8B,F) exposure led to a higher incidence of metastatic HCC. This suggests that *twist1a*, *xmrk,* LPS, and DSS can interact with the immune system and thereby participate in the development of tumor metastasis. This provides strong support of assertions indicating a relationship between chronic inflammation and tumor metastasis.

## 4. Materials and Methods

### 4.1. Zebrafish Husbandry

The fabp10a:mCherry-T2A-twist1a-ER^T2^ transgenic zebrafish line (abbreviated as fabp10a-twist1a or *twist1a+*), previously generated using the maize Activator (Ac)/Dissociation (Ds) transposon system, expresses hepatocyte-specific *twist1a* [30,64]. The previously generated fabp10a:TA; TRE:xmrk; krt4:GFP (abbreviated as fabp10a:xmrk or *xmrk+*) transgenic fish line expresses the hepatocyte-specific *xmrk* [31]. Wild-type control, Tg(fabp10a-twist1a), Tg(fabp10a:xmrk), Tg(lyz:DsRed), and Tg(mpeg1:mCherry) zebrafish embryos and larvae were maintained in E3 medium. Adult zebrafish were maintained at 28 °C under continuous flow under a 14-h light/10-h dark cycle [30,65]. This study was conducted in accordance with the Guide for the Care and Use of Laboratory Animals of the National Institutes of Health. All experiments involving zebrafish were approved by the Institutional Animal Care and Use Committee (IACUC) of the National University of Singapore and National Taiwan University.

### 4.2. Generation of fabp10a:twist1a/xmrk Double Transgenic Zebrafish

To establish a fabp10a:twist1a/xmrk (abbreviated as *twist1a+/xmrk+*) double transgenic zebrafish, we crossed Tg(fabp10a-twist1a) and Tg (fabp10a:xmrk) and then selected larvae positive for both transgenes for further study. Positive F1 embryos from *twist1a+/xmrk+* double transgenic zebrafish were maintained under the zebrafish husbandry conditions described above, until reaching the adult stage.

### 4.3. Isolation of RNA and Reverse-Transcription-PCR (RT-PCR)

Total RNA was isolated from the primary liver tumor, metastatic liver tumors, and adjacent normal tissue using the RNeasy Mini Kit (Qiagen, Hilden, Germany). RNA (1 μg) was then reverse transcribed into cDNA using the QuantiTect Whole Transcriptome Kit (Qiagen, Hilden, Germany). Following reverse transcription, cDNA templates were amplified via polymerase chain reaction (PCR) using exTEN 2X PCR Master Mix (Axil Scientific, Singapore). The primer sequences of the liver markers and internal control used for RT-PCR were as follows: *fabp10a* (Forward: CCAGTGACAGAAATCCAGCA; Reverse: GTTCTGCAGACCAGCTTTCC), *tfa* (Forward: TGCAGAAAAAGCTGGTGATG; Reverse: ACAGCATGAACTGGCACTTG), and *actin* (Forward: CTCCATCATGAAGTGCGACGT; Reverse: CAGACGGAGTATTTGCGCTCA). In the RT-PCR reaction, 1 µL of cDNA was amplified using the following protocol: 1 cycle at 95 °C for 5 min, followed by 35 cycles at 95 °C for 10 s, 58 °C for 30 s, and 68 °C for 1 min, eventually followed by incubation at 68 °C for an additional 7 min to allow for synthesis completion. Assaying the cDNA involved subjecting the PCR product to 1.0% agarose gel electrophoresis, using actin as an internal control.

### 4.4. Induction of Transgene Expression using Doxycycline and 4-Hydroxytamoxifen

Transgenic larvae were screened for fluorescence at 5 days post-fertilization (dpf) using a fluorescence stereo microscope (SMZ18, Nikon, Japan). The larvae were sorted according to whether EGFP and/or mCherry fluorescence was detected. The induction study was conducted on adult fish (3 to 4 months post-fertilization; mpf) in 5-L tanks with fresh water replenished every other day. Doxycycline (Dox, Sigma-Aldrich, St Louis, MO, USA) was used for the induction of *xmrk*, and 4-Hydroxytamoxifen (4-OHT, Sigma-Aldrich, St Louis, MO, USA) was used for the induction of *twist1a*. Long-term liver tumor metastasis induction involved treating *twist1a+*, *xmrk+*, and *twist1a+/xmrk+* transgenic zebrafish as well as their wild-type siblings using 30 (low dose) or 60 (high dose) μg/mL Dox and 0.5 or 1 μg/mL 4-OHT for 2, 4, 5, 6, or 8 weeks to maintain tumor growth and induce metastasis.

### 4.5. Induction of Transgene Expression, and Chemical Exposure to Transgenic Zebrafish

We sought to determine whether inflammatory agents DSS (Catalog number: D8906; Sigma-Aldrich, St. Louis, MO, USA) or LPS (Catalog number: L4391; Sigma-Aldrich, St. Louis, MO, USA) could cause inflammation in 4-day-old lyz:DsRed and mpeg1:mCherry transgenic zebrafish larvae. Each exposure group included 30 larvae maintained in 6-well plates containing 1 × E3 medium and 0.05% DSS or 40 ng/mL LPS for a period of 2 or 3 days.

We chemically induced *xmrk* expression by maintaining groups of *xmrk+* transgenic larvae (*n* = 20) in 6-well plates containing 1 × E3 medium, 20 μg/mL Dox, and 0.05% DSS or 40 ng/mL LPS for 3 days post-induction (dpi). For DSS or LPS treatment groups, zebrafish were treated with 20 μg/mL Dox as well as LPS 40 ng/mL or DSS 0.00625%. To induce *twist1a* or *xmrk* expression, zebrafish were, respectively, exposed to 1μg/mL 4-OHT or 20 μg/mL Dox for 2 wpi.

The double expression of *twist1a/xmrk* was induced via exposure to LPS or DSS in 5-L tanks at room temperature. Each treatment group was treated using 60 μg/mL Dox and 1μg/mL 4-OHT with LPS 40 ng/mL or DSS 0.00625%. The double expression of *twist1a/xmrk* was induced via exposure to 60 μg/mL Dox with 1 μg/mL 4-OHT for 4 wpi.

In this set of experiments, all larvae were maintained in 6-well plates containing 1 × E3 medium. After reaching the adult stage, the zebrafish were maintained in 5-L tanks at room temperature. Fresh water, Dox, 4-OHT, LPS, and DSS were replenished every other day. Samples were collected to investigate long-term treatment effects, and the mortality of adult zebrafish was estimated daily.

### 4.6. Collection of Tissue and Immunohistochemistry Staining

Tissue samples were collected from zebrafish following euthanization at 2, 4, 5, 6, or 8 wpi. Liver tissues were fixed and embedded in paraffin for histological and immunohistochemistry analysis, as previously described [45,46]. The 5-mmicrometer sections were deparaffined, rehydrated, and then treated with 3% H_2_O_2_ to block endogenous peroxidase activity, followed by heating in 10 mM citrate buffer at 100 °C for 20 min for antigen retrieval. Immunohistochemical analysis was performed with EnVision™+ Dual Link System (Dako, Carpinteria, CA, USA). Slides were treated using Dako peroxidase block buffer for 15 min and then incubated in primary antibodies at 4 °C overnight. The primary antibodies included rabbit anti-PCNA (1:500 dilutions; Catalog Number: FL-261, Santa Cruz, CA, USA), rabbit anti-caspase-3 (1:200 dilutions; Catalog Number: C92-065, BD Biosciences, USA), mouse anti-E-cadherin (1:200 dilutions; Catalog Number: 610188, BD Biosciences, San Diego, CA, USA), and mouse anti-Vimentin (1:200 dilutions; Catalog Number: 610188, Abcam, Cambridge, MA, USA). After washing with 1× PBS, slides were washed in 1x PBS with 0.1% Tween 20, developed with Dako DAB staining buffer, counterstained with hematoxylin before being dehydrated, cleared, and mounted with slide covers for evaluation using an Axio Imager Z2 microscope (Zeiss, Carl Zeiss Meditec AG, Germany).

### 4.7. Statistical Analysis

All statistical analysis in this study involved comparisons between experimental and control groups using one-way analysis of variance (ANOVA) and a two-tailed unpaired Student’s *t*-tests. Kaplan–Meier survival curves and log-rank tests were performed using GraphPad Prism 9 (GraphPad Software, La Jolla, CA, USA) as previously described [45,46]. *p*-values of 0.05 or less were considered statistically significant.

## 5. Conclusions

In conclusion, our results identify *Twist1* as an effective target gene against human HCC metastasis. This study provides the first in vivo demonstration that *twist1a* plays a critical role in both the maintenance and acceleration of *xmrk*-induced liver tumor metastasis in adult-stage zebrafish. We generated a novel autochthonous transgenic zebrafish model to demonstrate that *twist1a and xmrk* overexpression cooperates with inflammatory agents to accelerate the onset of tumor metastasis.

## Data Availability

Data are contained within the article.

## Supplementary Materials

The following are available online at www.mdpi.com/article/10.3390/ph14090867/s1, Figure S1: Fluorescent images of *twist1a*+ transgenic zebrafish and a timeline of experimental design with the schedule used for specimen collection; Figure S2: Liver tumor status and metastasis in *xmrk*+ and *twist1a*+/*xmrk*+ transgenic zebrafish.

## References

[1] Lu J.W., Chang J.G., Yeh K.T., Chen R.M., Tsai J.J., Hu R.M. (2011). Decreased expression of p39 is associated with a poor prognosis in human hepatocellular carcinoma. Med. Oncol..

[2] Lu J.W., Ho Y.J., Yang Y.J., Liao H.A., Ciou S.C., Lin L.I., Ou D.L. (2015). Zebrafish as a disease model for studying human hepatocellular carcinoma. World J. Gastroenterol..

[3] Siegel R.L., Miller K.D., Jemal A. (2018). Cancer statistics, 2018. CA Cancer J. Clin..

[4] Benson A.B., D’Angelica M.I., Abbott D.E., Abrams T.A., Alberts S.R., Saenz D.A., Are C., Brown D.B., Chang D.T., Covey A.M. (2017). NCCN Guidelines Insights: Hepatobiliary Cancers, Version 1.2017. J. Natl. Compr. Cancer Netw..

[5] Marks E.I., Yee N.S. (2016). Molecular Genetics and Targeted Therapy in Hepatocellular Carcinoma. Curr. Cancer Drug Targets.

[6] El-Serag H.B., Mason A.C., Key C. (2001). Trends in survival of patients with hepatocellular carcinoma between 1977 and 1996 in the United States. Hepatology.

[7] Valastyan S., Weinberg R.A. (2011). Tumor metastasis: Molecular insights and evolving paradigms. Cell.

[8] Kalluri R., Weinberg R.A. (2009). The basics of epithelial-mesenchymal transition. J. Clin. Investig..

[9] Nieto M.A. (2013). Epithelial plasticity: A common theme in embryonic and cancer cells. Science.

[10] Thiery J.P., Acloque H., Huang R.Y., Nieto M.A. (2009). Epithelial-mesenchymal transitions in development and disease. Cell.

[11] Puisieux A., Brabletz T., Caramel J. (2014). Oncogenic roles of EMT-inducing transcription factors. Nat. Cell Biol..

[12] Beck B., Lapouge G., Rorive S., Drogat B., Desaedelaere K., Delafaille S., Dubois C., Salmon I., Willekens K., Marine J.C. (2015). Different levels of Twist1 regulate skin tumor initiation, stemness, and progression. Cell Stem Cell.

[13] Mani S.A., Guo W., Liao M.J., Eaton E.N., Ayyanan A., Zhou A.Y., Brooks M., Reinhard F., Zhang C.C., Shipitsin M. (2008). The epithelial-mesenchymal transition generates cells with properties of stem cells. Cell.

[14] Morel A.P., Lievre M., Thomas C., Hinkal G., Ansieau S., Puisieux A. (2008). Generation of breast cancer stem cells through epithelial-mesenchymal transition. PLoS ONE.

[15] Chen Z.F., Behringer R.R. (1995). twist is required in head mesenchyme for cranial neural tube morphogenesis. Genes Dev..

[16] Ansieau S., Morel A.P., Hinkal G., Bastid J., Puisieux A. (2010). TWISTing an embryonic transcription factor into an oncoprotein. Oncogene.

[17] Entz-Werle N., Stoetzel C., Berard-Marec P., Kalifa C., Brugiere L., Pacquement H., Schmitt C., Tabone M.D., Gentet J.C., Quillet R. (2005). Frequent genomic abnormalities at TWIST in human pediatric osteosarcomas. Int. J. Cancer.

[18] Kwok W.K., Ling M.T., Lee T.W., Lau T.C., Zhou C., Zhang X., Chua C.W., Chan K.W., Chan F.L., Glackin C. (2005). Up-regulation of TWIST in prostate cancer and its implication as a therapeutic target. Cancer Res..

[19] Ohuchida K., Mizumoto K., Ohhashi S., Yamaguchi H., Konomi H., Nagai E., Yamaguchi K., Tsuneyoshi M., Tanaka M. (2007). Twist, a novel oncogene, is upregulated in pancreatic cancer: Clinical implication of Twist expression in pancreatic juice. Int. J. Cancer.

[20] Gort E.H., van Haaften G., Verlaan I., Groot A.J., Plasterk R.H., Shvarts A., Suijkerbuijk K.P., van Laar T., van der Wall E., Raman V. (2008). The TWIST1 oncogene is a direct target of hypoxia-inducible factor-2alpha. Oncogene.

[21] Yang M.H., Wu M.Z., Chiou S.H., Chen P.M., Chang S.Y., Liu C.J., Teng S.C., Wu K.J. (2008). Direct regulation of TWIST by HIF-1alpha promotes metastasis. Nat. Cell Biol..

[22] Da Silva S.D., Alaoui-Jamali M.A., Soares F.A., Carraro D.M., Brentani H.P., Hier M., Rogatto S.R., Kowalski L.P. (2014). TWIST1 is a molecular marker for a poor prognosis in oral cancer and represents a potential therapeutic target. Cancer.

[23] Ohba K., Miyata Y., Matsuo T., Asai A., Mitsunari K., Shida Y., Kanda S., Sakai H. (2014). High expression of Twist is associated with tumor aggressiveness and poor prognosis in patients with renal cell carcinoma. Int. J. Clin. Exp. Pathol..

[24] Yeo S.Y., Ha S.Y., Lee K.W., Cui Y., Yang Z.T., Xuan Y.H., Kim S.H. (2017). Twist1 is highly expressed in cancer-associated fibroblasts of esophageal squamous cell carcinoma with a prognostic significance. Oncotarget.

[25] Yusup A., Huji B., Fang C., Wang F., Dadihan T., Wang H.J., Upur H. (2017). Expression of trefoil factors and TWIST1 in colorectal cancer and their correlation with metastatic potential and prognosis. World J. Gastroenterol..

[26] Yang J., Mani S.A., Donaher J.L., Ramaswamy S., Itzykson R.A., Come C., Savagner P., Gitelman I., Richardson A., Weinberg R.A. (2004). Twist, a master regulator of morphogenesis, plays an essential role in tumor metastasis. Cell.

[27] Morel A.P., Hinkal G.W., Thomas C., Fauvet F., Courtois-Cox S., Wierinckx A., Devouassoux-Shisheboran M., Treilleux I., Tissier A., Gras B. (2012). EMT inducers catalyze malignant transformation of mammary epithelial cells and drive tumorigenesis towards claudin-low tumors in transgenic mice. PLoS Genet.

[28] Tsai J.H., Donaher J.L., Murphy D.A., Chau S., Yang J. (2012). Spatiotemporal regulation of epithelial-mesenchymal transition is essential for squamous cell carcinoma metastasis. Cancer Cell.

[29] Smit M.A., Peeper D.S. (2008). Deregulating EMT and senescence: Double impact by a single twist. Cancer Cell.

[30] Nakayama J., Lu J.W., Makinoshima H., Gong Z. (2020). A Novel Zebrafish Model of Metastasis Identifies the HSD11beta1 Inhibitor Adrenosterone as a Suppressor of Epithelial-Mesenchymal Transition and Metastatic Dissemination. Mol. Cancer Res..

[31] Li Z., Huang X., Zhan H., Zeng Z., Li C., Spitsbergen J.M., Meierjohann S., Schartl M., Gong Z. (2012). Inducible and repressable oncogene-addicted hepatocellular carcinoma in Tet-on xmrk transgenic zebrafish. J. Hepatol..

[32] Fausto N. (1999). Mouse liver tumorigenesis: Models, mechanisms, and relevance to human disease. Semin. Liver Dis..

[33] Lewis B.C., Klimstra D.S., Socci N.D., Xu S., Koutcher J.A., Varmus H.E. (2005). The absence of p53 promotes metastasis in a novel somatic mouse model for hepatocellular carcinoma. Mol. Cell. Biol..

[34] Fouad Y.A., Aanei C. (2017). Revisiting the hallmarks of cancer. Am. J. Cancer Res..

[35] Yan C., Yang Q., Huo X., Li H., Zhou L., Gong Z. (2017). Chemical inhibition reveals differential requirements of signaling pathways in kras(V12)- and Myc-induced liver tumors in transgenic zebrafish. Sci. Rep..

[36] Hoffman B., Liebermann D.A. (2008). Apoptotic signaling by c-MYC. Oncogene.

[37] Hognason T., Chatterjee S., Vartanian T., Ratan R.R., Ernewein K.M., Habib A.A. (2001). Epidermal growth factor receptor induced apoptosis: Potentiation by inhibition of Ras signaling. FEBS Lett..

[38] Coffelt S.B., Wellenstein M.D., de Visser K.E. (2016). Neutrophils in cancer: Neutral no more. Nat. Rev. Cancer.

[39] Wu L., Zhang X.H. (2020). Tumor-Associated Neutrophils and Macrophages-Heterogenous but Not Chaotic. Front. Immunol..

[40] Tahmasebi Birgani M., Carloni V. (2017). Tumor Microenvironment, a Paradigm in Hepatocellular Carcinoma Progression and Therapy. Int. J. Mol. Sci..

[41] De Giorgio M., Fagiuoli S. (2007). Management of hepatocellular carcinoma. Dig. Dis..

[42] Lamouille S., Xu J., Derynck R. (2014). Molecular mechanisms of epithelial-mesenchymal transition. Nat. Rev. Mol. Cell. Biol..

[43] Li B., Han Q., Zhu Y., Yu Y., Wang J., Jiang X. (2012). Down-regulation of miR-214 contributes to intrahepatic cholangiocarcinoma metastasis by targeting Twist. FEBS J..

[44] Zhu S., Zhang X., Weichert-Leahey N., Dong Z., Zhang C., Lopez G., Tao T., He S., Wood A.C., Oldridge D. (2017). LMO1 Synergizes with MYCN to Promote Neuroblastoma Initiation and Metastasis. Cancer Cell.

[45] Nguyen A.T., Emelyanov A., Koh C.H., Spitsbergen J.M., Parinov S., Gong Z. (2012). An inducible kras(V12) transgenic zebrafish model for liver tumorigenesis and chemical drug screening. Dis. Model. Mech..

[46] Chew T.W., Liu X.J., Liu L., Spitsbergen J.M., Gong Z., Low B.C. (2014). Crosstalk of Ras and Rho: Activation of RhoA abates Kras-induced liver tumorigenesis in transgenic zebrafish models. Oncogene.

[47] Che N., Zhao X.L., Sun T., Zhao X.M., Gu Q., Dong X.Y., Zhao N., Liu Y.R., Yao Z., Sun B.C. (2011). The role of Twist1 in hepatocellular carcinoma angiogenesis: A clinical study. Hum. Pathol..

[48] Schneider M.R., Hiltwein F., Grill J., Blum H., Krebs S., Klanner A., Bauersachs S., Bruns C., Longerich T., Horst D. (2014). Evidence for a role of E-cadherin in suppressing liver carcinogenesis in mice and men. Carcinogenesis.

[49] Calvisi D.F., Ladu S., Conner E.A., Factor V.M., Thorgeirsson S.S. (2004). Disregulation of E-cadherin in transgenic mouse models of liver cancer. Lab. Investig..

[50] Chen J., Zhao J., Ma R., Lin H., Liang X., Cai X. (2014). Prognostic significance of E-cadherin expression in hepatocellular carcinoma: A meta-analysis. PLoS ONE.

[51] Han L.L., Jia L., Wu F., Huang C. (2019). Sirtuin6 (SIRT6) Promotes the EMT of Hepatocellular Carcinoma by Stimulating Autophagic Degradation of E-Cadherin. Mol. Cancer Res..

[52] Ivaska J., Pallari H.M., Nevo J., Eriksson J.E. (2007). Novel functions of vimentin in cell adhesion, migration, and signaling. Exp. Cell Res..

[53] Ivaska J., Vuoriluoto K., Huovinen T., Izawa I., Inagaki M., Parker P.J. (2005). PKCepsilon-mediated phosphorylation of vimentin controls integrin recycling and motility. EMBO J..

[54] Kokkinos M.I., Wafai R., Wong M.K., Newgreen D.F., Thompson E.W., Waltham M. (2007). Vimentin and epithelial-mesenchymal transition in human breast cancer–Observations in vitro and in vivo. Cells Tissues Organs.

[55] Hu L., Lau S.H., Tzang C.H., Wen J.M., Wang W., Xie D., Huang M., Wang Y., Wu M.C., Huang J.F. (2004). Association of Vimentin overexpression and hepatocellular carcinoma metastasis. Oncogene.

[56] Hendrix M.J., Seftor E.A., Seftor R.E., Trevor K.T. (1997). Experimental co-expression of vimentin and keratin intermediate filaments in human breast cancer cells results in phenotypic interconversion and increased invasive behavior. Am. J. Pathol..

[57] Neve R.M., Chin K., Fridlyand J., Yeh J., Baehner F.L., Fevr T., Clark L., Bayani N., Coppe J.P., Tong F. (2006). A collection of breast cancer cell lines for the study of functionally distinct cancer subtypes. Cancer Cell.

[58] Sun S., Poon R.T., Lee N.P., Yeung C., Chan K.L., Ng I.O., Day P.J., Luk J.M. (2010). Proteomics of hepatocellular carcinoma: Serum vimentin as a surrogate marker for small tumors (<or=2 cm). J. Proteome Res..

[59] Makol A., Kaur H., Sharma S., Kanthaje S., Kaur R., Chakraborti A. (2020). Vimentin as a potential therapeutic target in sorafenib resistant HepG2, a HCC model cell line. Clin. Mol. Hepatol..

[60] Chang Y.S., Chen W.Y., Yin J.J., Sheppard-Tillman H., Huang J., Liu Y.N. (2015). EGF Receptor Promotes Prostate Cancer Bone Metastasis by Downregulating miR-1 and Activating TWIST1. Cancer Res..

[61] Tran P.T., Shroff E.H., Burns T.F., Thiyagarajan S., Das S.T., Zabuawala T., Chen J., Cho Y.J., Luong R., Tamayo P. (2012). Twist1 suppresses senescence programs and thereby accelerates and maintains mutant Kras-induced lung tumorigenesis. PLoS Genet..

[62] Lu J.W., Raghuram D., Fong P.A., Gong Z. (2018). Inducible Intestine-Specific Expression of kras(V12) Triggers Intestinal Tumorigenesis in Transgenic Zebrafish. Neoplasia.

[63] Yan C., Huo X., Wang S., Feng Y., Gong Z. (2015). Stimulation of hepatocarcinogenesis by neutrophils upon induction of oncogenic kras expression in transgenic zebrafish. J. Hepatol..

[64] Emelyanov A., Gao Y., Naqvi N.I., Parinov S. (2006). Trans-kingdom transposition of the maize dissociation element. Genetics.

[65] Lu J.W., Hou H.A., Hsieh M.S., Tien H.F., Lin L.I. (2016). Overexpression of FLT3-ITD driven by spi-1 results in expanded myelopoiesis with leukemic phenotype in zebrafish. Leukemia.

