# Supplementary material for: Exacerbation of Liver Tumor Metastasis in twist1a+/xmrk+ Double Transgenic Zebrafish following Lipopolysaccharide or Dextran Sulphate Sodium Exposure
Source: Pharmaceuticals (Basel). 2021 Aug 28;14(9):867. doi: 10.3390/ph14090867 (PMC8468836; doi:10.3390/ph14090867)
Supplement: Supplementary file 1 [file pharmaceuticals-14-00867-s001.zip › pharmaceuticals-1349431-supplementary.pdf]

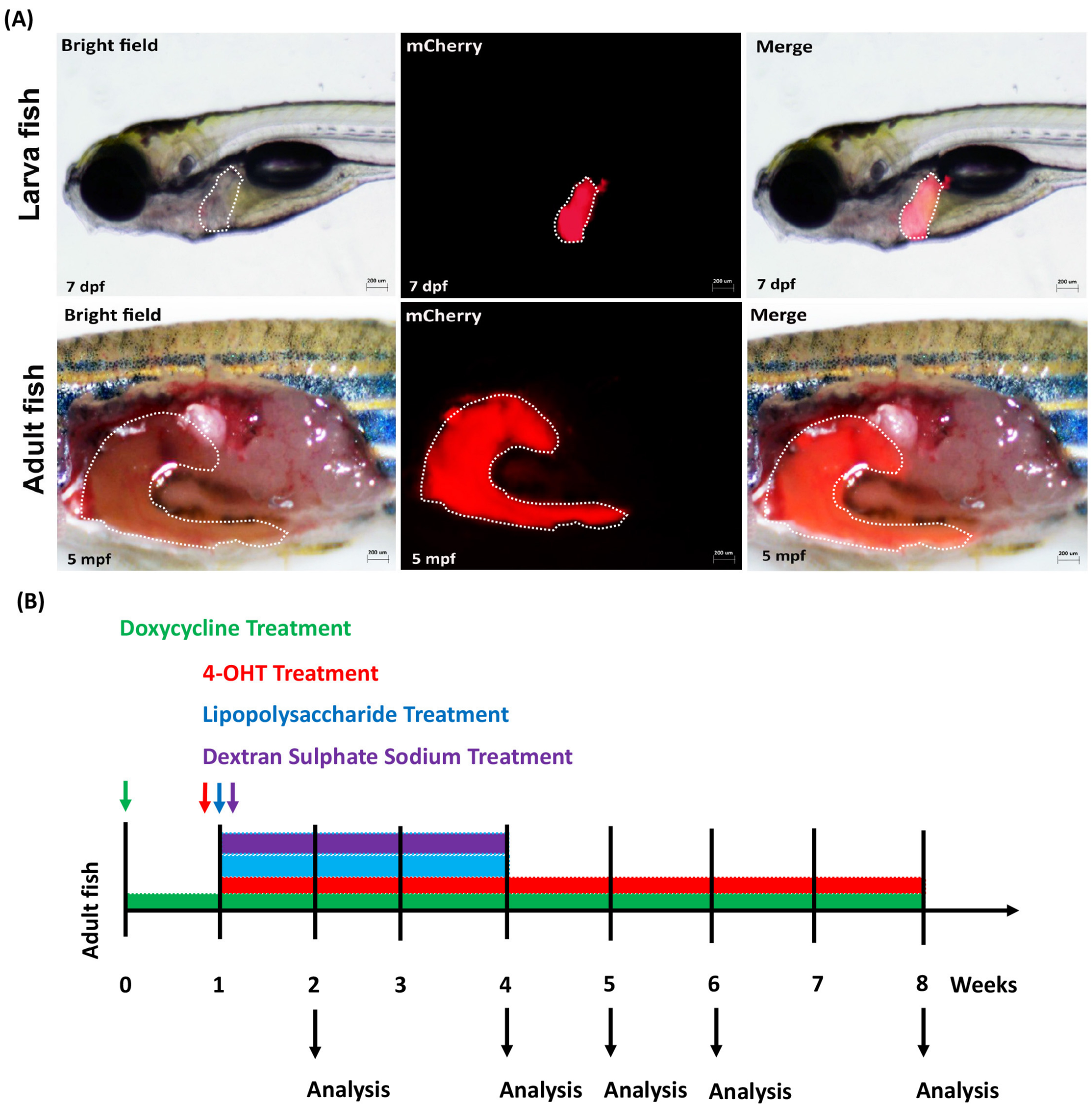

Figure S1. Fluorescent images of *twist1a*+ transgenic zebrafish and a timeline of experimental design with the schedule used for specimen collection. **(A)** Fluorescent images of *twist1a*+ transgenic larva at 7 dpf and adult zebrafish at 5 mpf. The red denotes fluorescent *twist1a*-expressing hepatocytes in the liver. **(B)** Experiment design and specimen collection schedule for long-term treatment.

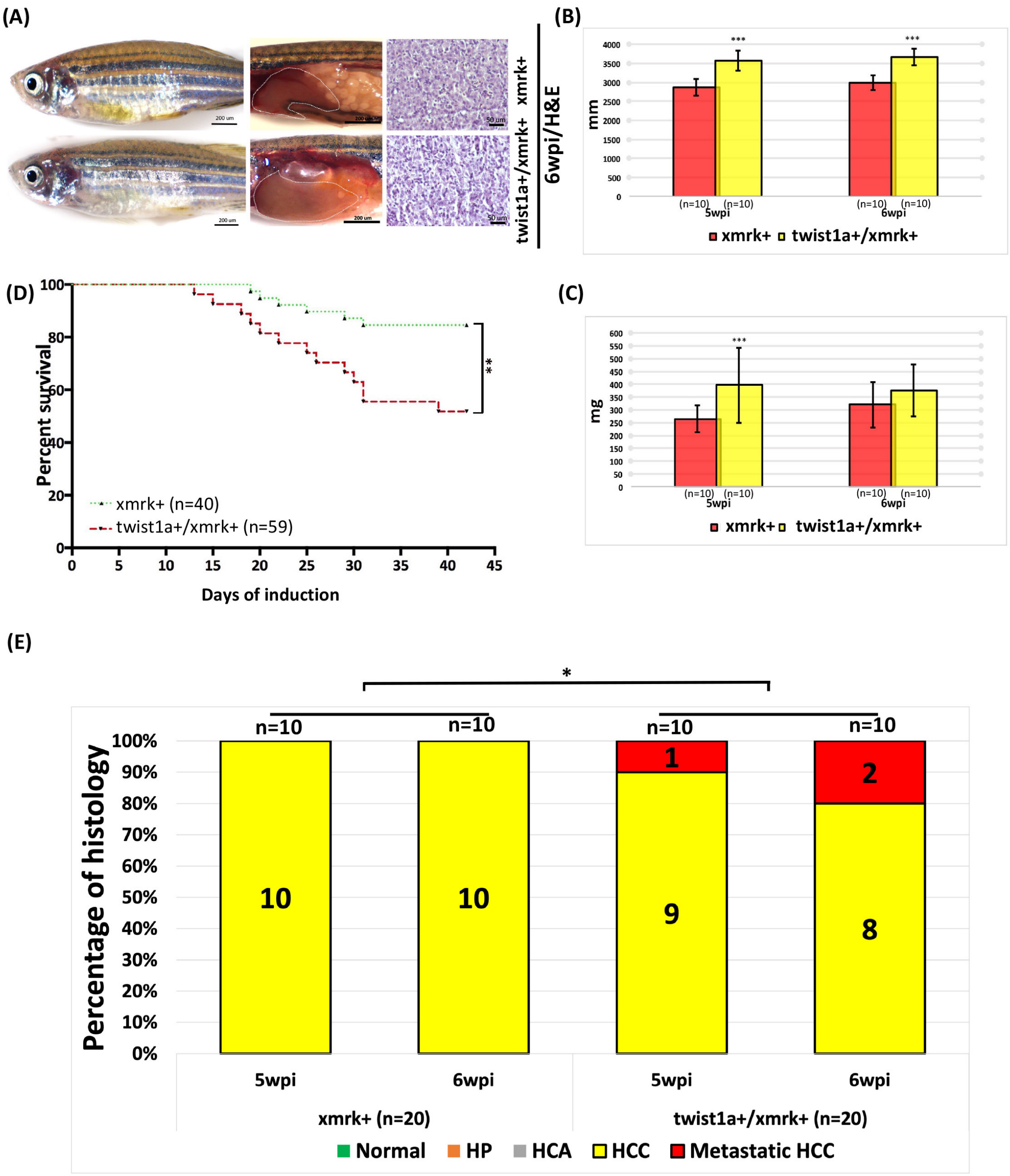

Figure S2. Liver tumor status and metastasis in *xmrk*+ and *twist1a*+/*xmrk*+ transgenic zebrafish: **(A)** *Twist1a*+/*xmrk*+ and *xmrk*+ control transgenic zebrafish were treated with 60 µg/ml Dox for three weeks, at which point the dose was reduced to 30 µg/ml Dox for a further three weeks. Note that 1 µg/ml 4-OHT was maintained throughout the course of the experiment to ensure the sustained induction of *twist1a*. Sampling was performed at 5 and 6 wpi. The left column displays the external appearance, the middle column shows internal abdominal organs with the livers outlined, and the right column depicts H&E staining of liver sections. Scale bar: 50 or 200 µm. In the *twist1a*+/*xmrk*+

transgenic zebrafish, we observed significant differences in **(B)** body length and **(C)** body weight at 5 and 6 wpi, compared with the *xmrk*+ group at the same timepoints. **(D)** Kaplan-Meier survival curves showing days post-induction plotted against percentage survival to 6 wpi. **(E)** Histological examination confirmed that *twist1a*+/*xmrk*+ transgenic zebrafish developed HCC or metastatic HCC at 5 and 6 wpi at an incidence exceeding that of *xmrk*+ siblings. Differences among the variables were assessed using Student's t-tests or one-way ANOVA. Statistical significance: *$P<0.05$, **$P<0.01$, ***$P<0.001$.
